# Super anticorrosion of aluminized steel by a controlled Mg supply

**DOI:** 10.1038/s41598-018-22097-z

**Published:** 2018-02-28

**Authors:** Jae In Jeong, Ji Hoon Yang, Jae Hun Jung, Kyung Hwang Lee, Hye Jeong Kim, Yong Hwa Jung, Tae Yeob Kim, Myeong Hoon Lee, Sung Hwa Hwang, Ping Wu, Jae-Hun Kim, Sang Sub Kim

**Affiliations:** 1 Materials Solution Research Group, Research Institute of Industrial Science & Technology, 67 Cheongam-ro, Nam-gu Pohang, 37673 Republic of Korea; 2 Posco Smart Coating Technology-Dry Coating Project Dept., POSCO Gwangyang Research Lab., 20-26 Pokposarang-gil, Gwangyang, 57807 Republic of Korea; 3 Division of Marine Engineering, Korea Maritime & Ocean University, 727 Taejong-ro, Yeongdo-gu Busan, 49112 Republic of Korea; 4 Entropic Interface Group, Singapore University of Technology & Design, Singapore, 487372 Singapore; 5 Department of Materials Science and Engineering, Inha University, Incheon, 22212 Republic of Korea

## Abstract

The current anticorrosion strategy makes use of coatings to passively protect the steel, which faces increasing challenge due to the tightened environmental regulations and high cost. This paper reports a new method for achieving a super anticorrosion function in Al-Si alloys through Mg nano-metallurgy, which was characterized by real-time synchrotron measurements. The unique function is based on the formation of an amorphous and self-charge-compensated MgAl_2_O_4_-SiO_2_ phase between the grain boundaries to help prevent the penetration of oxygen species through the grain boundaries. Through this, the corrosion resistance of pristine aluminized steel could be improved almost 20 fold. An analysis of the phases, microstructures of the Mg-coated aluminized layer and corrosion products consistently supported the proposed mechanism. This charge-compensated corrosion resistance mechanism provides novel insight into corrosion resistance.

## Introduction

The cost of corrosion in modern industrialized societies is enormous, making its suppression an ongoing and challenging issue^1–3^. Therefore, this paper reports a new method to produce surface nano-structures by nano-metallurgy that block the penetration of oxygen species through the grain boundaries.

Hot-dip galvanized steel has been used widely in a variety of applications for more than 100 years because of its superb durability and corrosion resistance^4,5^. In recent years, there has been extensive research into improving its corrosion resistance^6–8^. As an alternative, hot-dip aluminized steel is used widely, particularly for materials in automotive exhaustion parts, outdoor building parts, and various high strength parts, on account of its superior corrosion resistance to hot-dip galvanized steel and its attractive surface^9–11^. The reason for the enhanced anti-oxidation capability of aluminized steel is the formation of a protective, dense, and stable Al_2_O_3_ layer on the surface of the aluminized layer on steels^12,13^.

The overall microstructure of the hot-dip aluminized steel can be categorized into two domains: an outer Al-Si layer and an inner intermetallic layer with the chemical compositions of Fe-Al-Si^13^. The intermetallic layer is believed to be a major source of corrosion and mechanical failure of aluminized steel, so a thick and loose intermetallic layer is not advantageous. For a thinner and flatter intermetallic layer, Si is added to the Al molten bath, usually at ~10 wt. % (close to the eutectic composition of 12 wt. %)^14–16^.

The mechanism of the reduction of the intermetallic layer by the addition of Si to the Al molten bath has been addressed in many studies^17,18^. In general, its hypoeutectic structure has 3 features: (1) primary α-Al (with ~2 wt. % Si), (2) eutectic Al (~2 wt. % Si), and (3) eutectic Si (no Al). Because Si is nobler than Al, when exposed to oxygen, the two α-Al features will be oxidized first followed by Si. Upon oxidation, all Al features lose electrons to form Al_2_O_3_, but with regards to the two α-Al features, the eutectic one is firstly fully covered with a protective Al_2_O_3_ layer due to its narrow thickness. The oxidation product of the Si layer is protective SiO_2_ that forms after the formation of Al_2_O_3_ in the structures. A primary Al particle is much larger and thicker; the Al_2_O_3_ coverage is not as homogeneous and dense as its eutectic counterpart. Therefore, among the different hypoeutectic structures, the observed corrosion rate decreases with increasing Si concentration, where the quantity of primary Al particles decreases^18^.

The bottleneck in the anticorrosion of Si-containing hot-dip aluminized steel is how to slow down the oxidation rate for the primary Al first, and then the eutectic Al layers. Most important, however, is how to prevent oxygen penetration through the loose interface between Al_2_O_3_ and SiO_2_. As will be discussed in the next section, chemically, clusters of Al_2_O_3_ and SiO_2_ prefer to be separate rather than form bonds, which provides an efficient and damaging path for the continuous supply of oxygen species towards the bulk from the surface. In this study, the self-charge-compensation mechanism, which is well known in glass making^19^, was used to transform the separated Al_2_O_3_ and SiO_2_ clusters into dense and locally ordered spinel-like MgAl_2_O_4_-SiO_2_ amorphous species during the oxidation process. This amorphous phase (like a glue) will help seal the oxygen path between the grains.

This paper reports the surprising improvement in the corrosion resistance in hot-dip aluminized (9 wt. % Si) steel by deliberately supplying Mg into the aluminized layer. The underlying mechanism based on the self-charge compensation for improving the corrosion resistance is proposed. In addition, the regulated supply of Mg was found to be essential for maximizing the role of Mg. This study is expected to pave the way for the development of a new paradigm in anticorrosion research and development.

## Results

### Microstructure and surface chemistry of the Mg-coated aluminized layer

**Figure 1:**
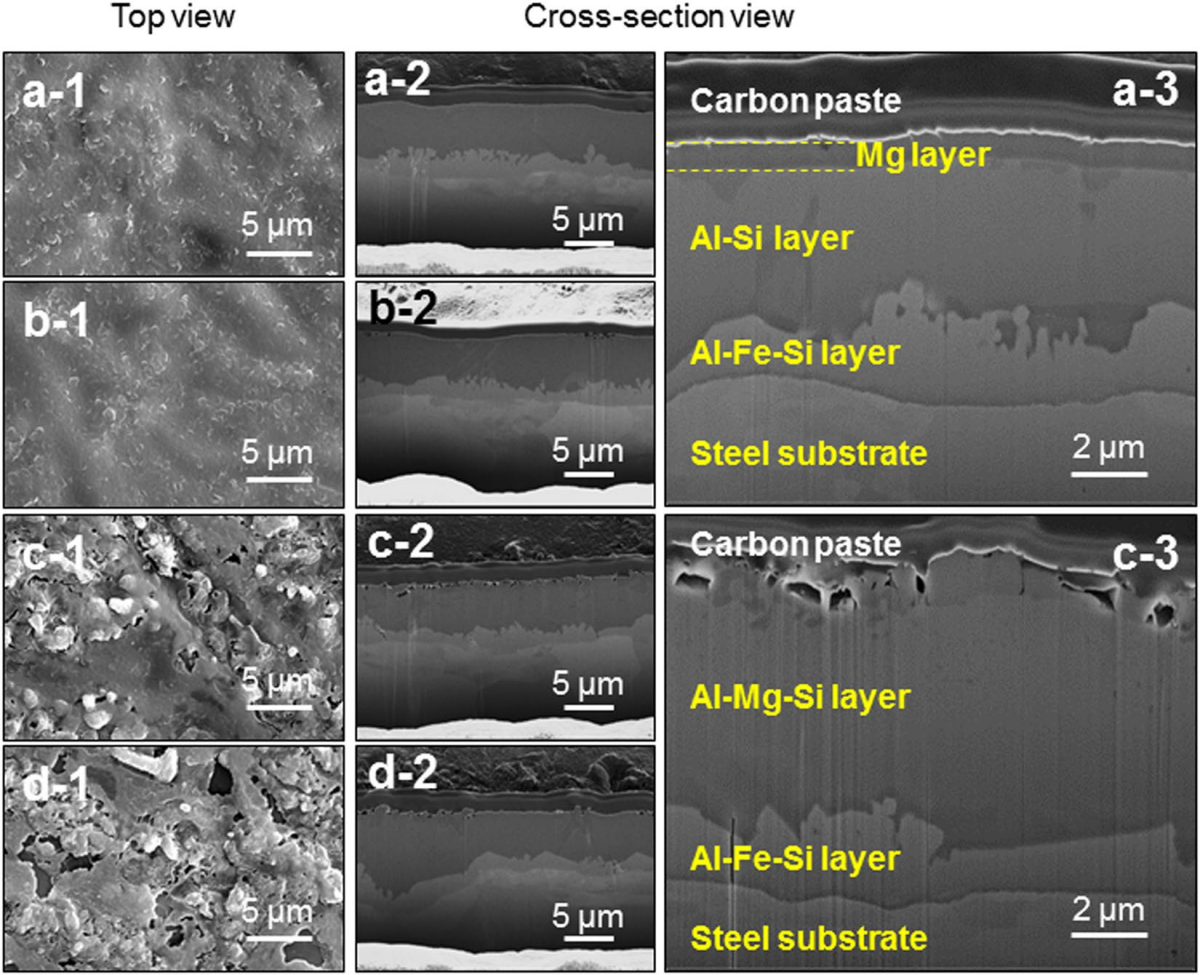
Scanning electron micrographs of the as-grown and heat-treated samples with a 0.5 μm-thick Mg layer: Top view and cross-section view images of (**a**–**1**) and (**a**–**2**) as-grown, (**b**–**1**) and (**b**–**2**) Type A, (**c**–**1**) and (**c**–**2**) Type B, and (**d**–**1**) and (**d**–**2**) Type C samples, respectively. (**a**–**3**) and (**c**–**3**) are magnified images for (**a**–**2**) and (**c**–**2**), respectively. Carbon paste was used as a conducting layer for observation.

**Figure 2:**
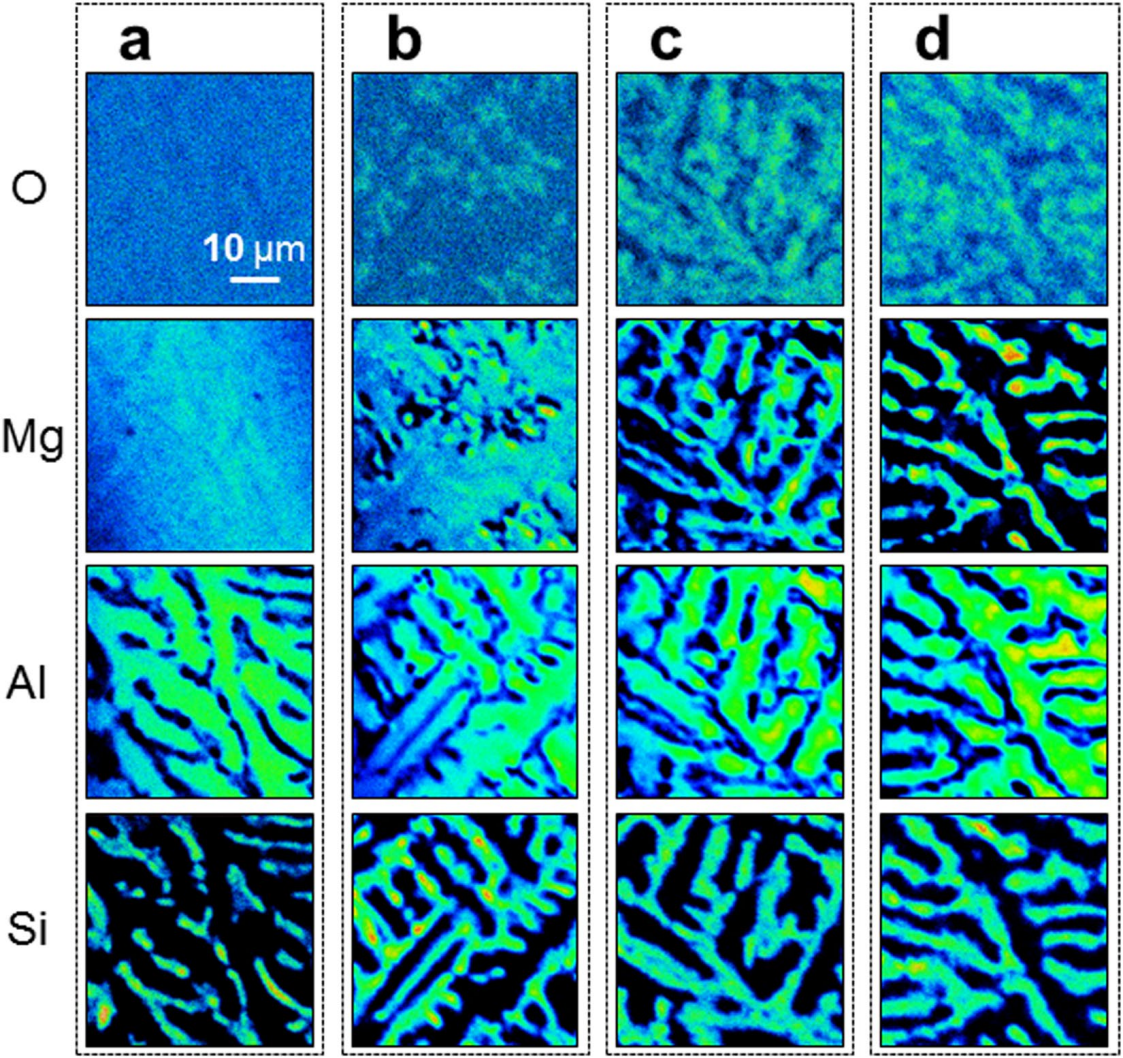
Top-view elemental maps by electron probe microanalysis for (**a**) as-grown, (**b**) Type A, (**c**) Type B, and (**d**) Type C samples with a 0.5 μm-thick Mg layer.

In the first place, the microstructures of the Mg-coated aluminized samples were investigated. Figure 1 presents SEM images of the surface and cross-section morphology of the as-grown and heat-treated samples with a 0.5 μm-thick Mg layer. As shown in Fig. 1(a–1),(a–2) and (a–3), before heat treatment, a 0.5 μm-thick Mg layer was deposited on the Al-Si eutectic lamellar structured layer. The rugged surface morphology is likely to originate from the evolution of a eutectic lamellar structure. After the initial stage of heat treatment, the Type A sample showed a somewhat smoother surface than the as-grown sample. During further heat treatment, the Type B and C samples exhibited a similar surface morphology to each other. The cross-section SEM images shown in Fig. 1(a–2),(a–3) reveal a distinctive Mg layer, aluminized layer, and intermetallic layer in sequence from the top. After heat treatment, the boundary between the Mg layer and aluminized layer disappeared, indicating severe inter-diffusion between the two layers, as shown in Fig. 1(c–2),(c–3). Similar behavior was observed in the samples with a 1.0 μm-thick Mg layer (*see* Supplementary Fig. 1).

**Figure 3:**
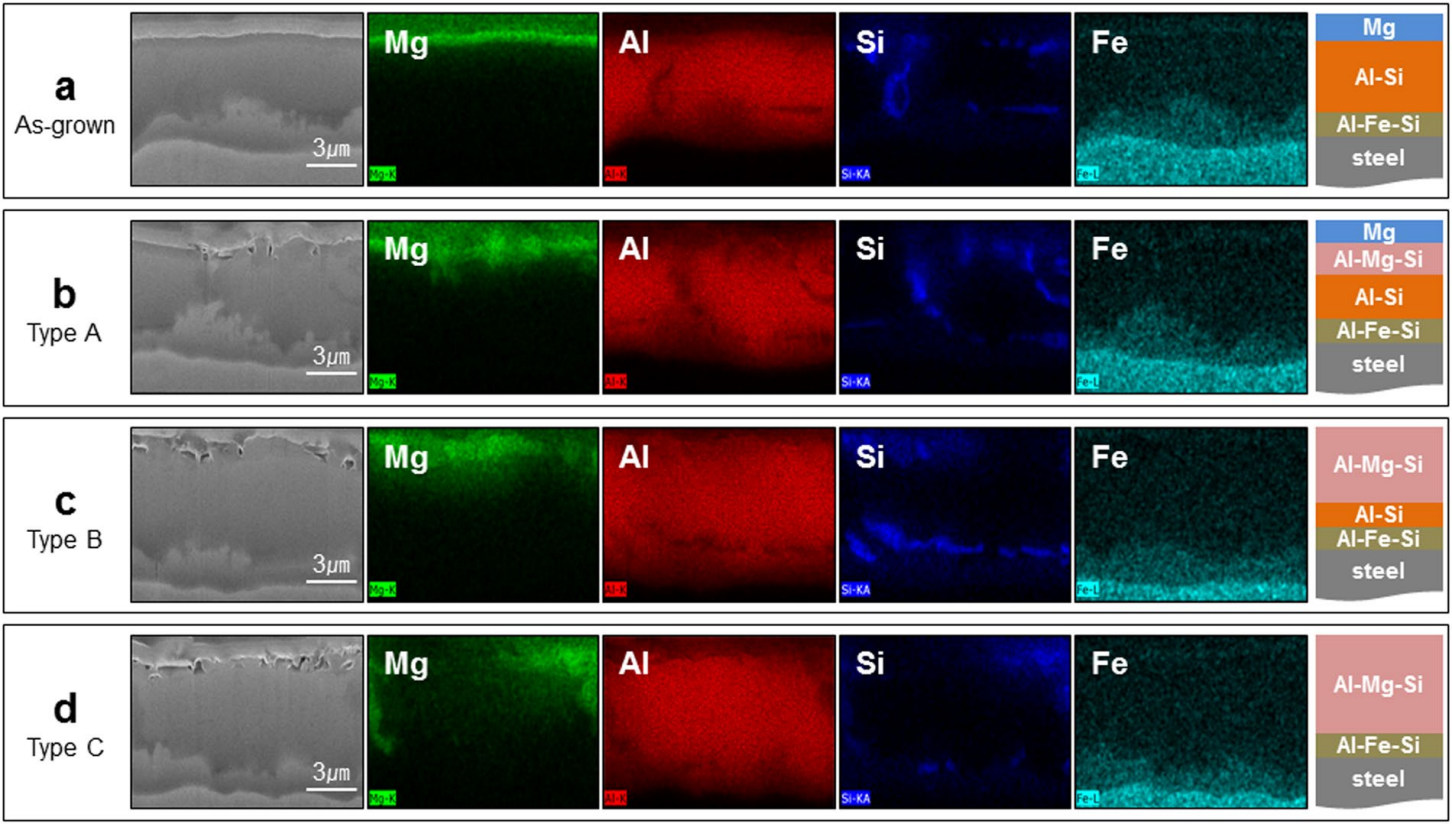
Cross-section view elemental maps by electron probe microanalysis for (**a**) as-grown, (**b**) Type A, (**c**) Type B, and (**d**) Type C samples with a 0.5 μm-thick Mg layer.

The observed surface morphology and chemistry evolution is also consistent with the water contact angle measurements. As shown in Supplementary Fig. 2, the water contact angles on the above 4 surfaces decreased from 41° on the as-grown Mg surface, to 11° on Type A, and then to 6 ± 1° on the Type B and C surfaces. In contrast, the water contact angle was approximately 75° on a smooth hypoeutectic Al-Si surface^18^, which increased with increasing surface roughness. Based on Wu’s entropy equation^20^, the above decrease in contact angle originates from the increase in entropy due to the formation of highly reactive surfaces. In contrast to conventional laws, the more hydrophilic surface with very low water contact angles (Types A, B, and C) was approximately 20 times more corrosion resistant than the more hydrophobic one with a water contact angle of 75° (*see* Section Corrosion behavior). The key to this unusual behavior is the formation of a self-charge-compensated phase, despite the much stronger water attachment to these surfaces.

Cross-section TEM was carried out for further microstructural analysis; typical results are summarized in Supplementary Figs 3 and 4. Regardless of the Mg layer thickness, the bright-field TEM images and corresponding elemental maps revealed the presence of a Mg layer on the top and the evolution of a grainy microstructure for the heat-treated sample, Type C. The distribution of Mg, Al, and Si was confined in the as-grown sample, but after heat treatment, these elements spread, indicating active interdiffusion.

The nature of the interdiffusion of the constituents in the aluminized layer during heat treatment can be understood qualitatively by examining the chemical composition. Figure 2 presents the top-view surface-region elemental maps by EPMA for the as-grown and A, B, and C samples with a 0.5 μm-thick Mg layer. A dynamic change in the distribution of each element was observed. Figure 3 presents the cross-section elemental maps. Before heat treatment, the Mg was confined to the surface region. Below the Mg layer, Al, and Si were distributed evenly, indicating the presence of a eutectic lamellar structure. During heat treatment, however, the significant interdiffusion of Mg, Al, and Si took place, forming intermetallic compounds, such as Al_3_Mg_2_, Al_12_Mg_17_, and Mg_2_Si, which were confirmed by X-ray diffraction (*see* Section Phase transformation). The same behavior was observed for the samples with a 1.0 μm-thick Mg layer (*see* Supplementary Fig. 6). In addition, the evolution of surface chemistry with regard to the heat treatment time was more pronounced in the thinner Mg-layer-coated sample. Supplementary Fig. 5 shows top-view elemental maps for the samples with a 0.3 μm-thick Mg layer, basically showing similar behavior to that shown in Fig. 2. This suggests that the evolution of surface chemistry is least dependent on the Mg layer thickness.

From the elemental mapping results in Figs 2 and 3, the following can be drawn for each stage. For the as-grown sample, oxygen distributes evenly over the surface, indicating that the Mg layer has been oxidized slightly before heat treatment. Mg is distributed evenly; that is, no reaction takes place and a thin film forms on the hot-dip aluminized Al-Si layer with a eutectic lamellar structure. In the elemental maps, the Al contrast originated mainly from the primary proeutectoid α-Al and partly from α-Al in the Al-Si eutectic structure. The Si contrast originates from Si in the eutectic structure.

For Type A, at the very initial stages of heat treatment, Mg forms islands, which suggests that the Mg_2_Si phase begins to form. In this case, the Al contrast originated from multi-sources, such as proeutectoid α-Al, Al_3_Mg_2_ (β), and Al_12_Mg_17_ (γ). On the other hand, the Si contrast originated mainly from the eutectic Si. From Fig. 3, Al is present near the top surface, even in the as-grown sample, whereas Si is located far below the Al layer. This is due partially to the relatively low Si to Al concentration ratio (1/9). In the Type A sample, Al diffused up and formed Al-Mg alloys (Al_3_Mg_2_ and Al_12_Mg_17_) while virtually no Mg_2_Si was detected. Moreover, the entropies at the water-air-alloy interfaces for Al_12_Mg_17_, Al_3_Mg_2_, and Mg_2_Si were 463, 478, and 487 J molar-mass^−1^ K^−1^, respectively^20^, because this entropy is a linear function of the water contact angle (low angle for high entropy and vice versa). Therefore, the above observation for the Type A sample is also supported by the measured water contact angles, which were 11°, 6 ± 1°, and 6 ± 1° for the Types A, B, and C samples, respectively. Among the 3 samples, the Type A sample had the highest Al_12_Mg_17_ content (because of its low entropy) (see Section Phase transformation). Therefore, at this stage of nano-metallurgy, Mg atoms are alloyed dominantly with an Al-rich phase and do not yet interact with Si to substantially form Mg-Si intermetallics. Consequently, at this nano-metallurgy stage, the structure is not ready to produce a self-charge-compensated amorphous MgAl_2_O_4_-SiO_2_ glue.

For type B, which was heat-treated further, the amount of Al_12_Mg_17_ is believed to decrease significantly. The phase boundary of Al is clearer than that in Type A because there are only two Al sources: primarily Al_3_Mg_2_ and a minor amount of proeutectoid α-Al. Interestingly, oxygen begins to show a pattern, which is mostly consistent with the Al component, so that the Al-Mg alloy is likely to oxidize first. This supports the formation of beneficial spinel MgAl_2_O_4_, which is thermodynamically favorable. Like the Fe-O system, where spinel Fe_3_O_4_ is denser and more oxidation resistant than the Fe_2_O_3_ phase, spinel MgAl_2_O_4_ provides better protection to the Al under layer than Al_2_O_3_. In addition, the O pattern partly reflects the Si component, suggesting the formation of SiO_2_. The Si pattern is in accordance with the Mg pattern, which is indicative of the significant formation of Mg_2_Si. Some parts of the Mg pattern are also consistent with the Al pattern because of the formation of Al_3_Mg_2_. Interestingly, at this stage of nano-metallurgy, Mg atoms are alloyed with both Al-rich and Si-rich phases, as shown in Fig. 2. This structure has the potential to nurture the desired amorphous glue phase because it provides both Al-Mg and Mg-Si bonds.

For Type C, the contrast arising from oxygen shows a more evident pattern, indicating an increase in the degree of oxide formation. Compared to Type B, the phase boundary of the Al pattern was much clearer because of (1) the absence of a Mg-rich phase (Al_12_Mg_17_) and (2) consumption of the Mg atoms by Si (to form Mg_2_Si) with little indication of Al-Mg bonds. The Si pattern is composed mainly of Mg_2_Si and SiO_2_. Compared to Type B, the pattern band becomes thicker because Mg_2_Si grows; Al_12_Mg_17_ dissolves and Mg in Al_12_Mg_17_ interacts with Si to form Mg_2_Si. At this nano-metallurgy stage, few Mg atoms remain in the Al_3_Mg_2_ phase but are dissolved mostly in the Mg_2_Si phase. The lack of Al-Mg bonds makes the formation of MgAl_2_O_4_ more difficult; consequently, an amorphous Mg-Al-Si-O glue phase forms between the grains.

### Phase transformation

**Figure 4:**
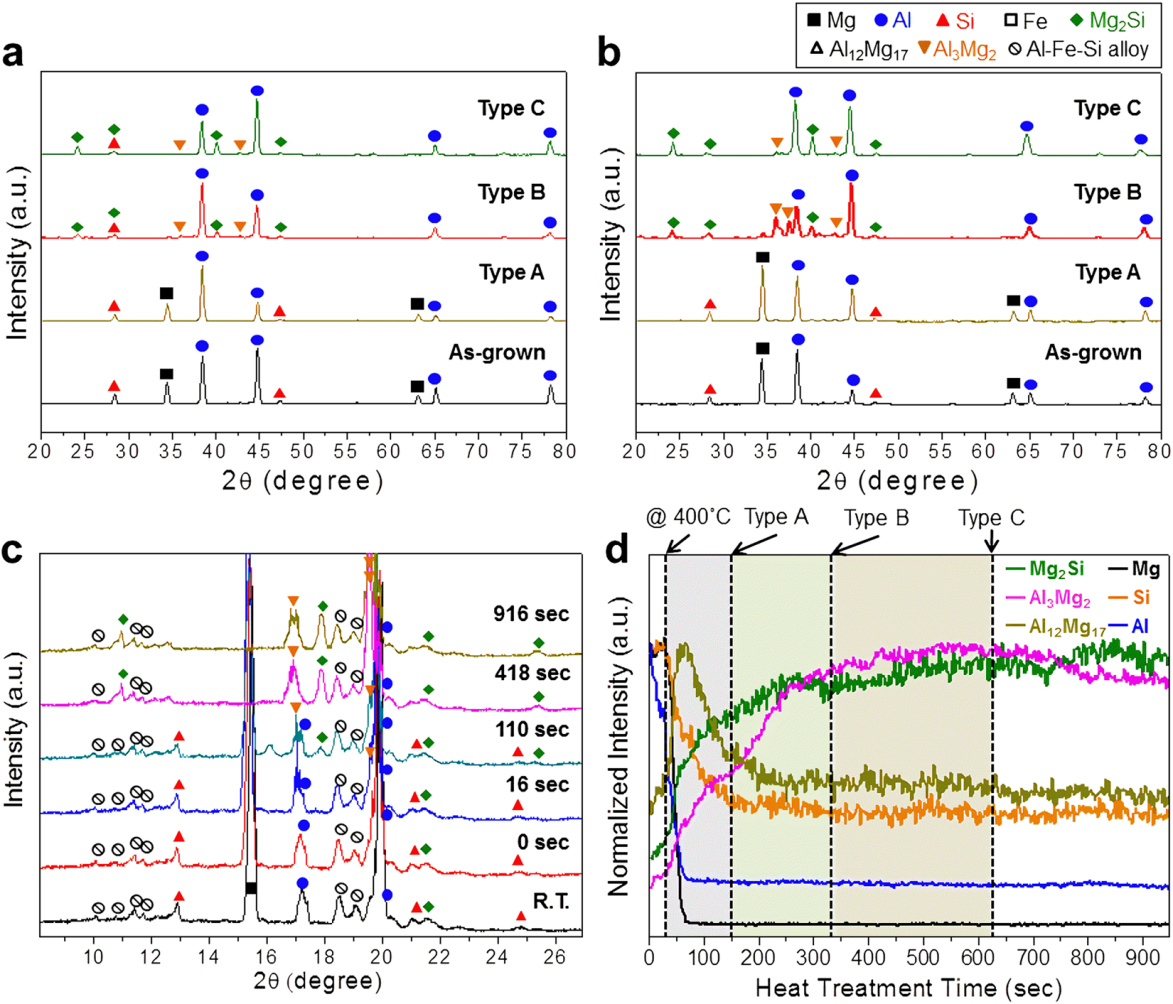
Representative X-ray diffraction patterns of the as-grown and heat-treated samples with (**a**) 0.5 μm-thick and (**b**) 1.0 μm-thick Mg layers. (**c**) A series of real-time synchrotron X-ray diffraction patterns with the heat treatment time at 400 °C, which were obtained with X-ray wavelength 1.757 Å. (**d**) Phase evolution during heat treatment based on real-time synchrotron X-ray diffraction.

The phase formation was investigated by XRD. Figure 4a,b shows representative XRD patterns of the as-grown and heat-treated samples with 0.5 μm-thick and 1.0 μm-thick Mg layers, respectively. In both images, the as-grown sample revealed the presence of Mg, Al, and Si phases without a noticeable intermetallic phase, meaning there was no substantial chemical reaction between the Mg layer and the Al-Si eutectic lamellar structure. As heat treatment proceeds, intermetallic phases, such as Mg_2_Si, Al_3_Mg_2_ (β), and Al_12_Mg_17_ (γ), begin to form, indicating the interdiffusion of the constituents, such as Al, Si, and Mg. The sharp enhancement of the corrosion resistance via the supply of Mg must be correlated with the formation of such intermetallic compounds.

Real-time XRD was carried out using synchrotron radiation to examine the kinetics of phase evolution. As shown in Fig. 4c, a series of XRD patterns were obtained for the samples with a 1.0 μm-thick Mg layer with the heat treatment time. The Bragg reflections with the highest intensity for each phase were selected to examine its formation. Figure 4d summarizes the phase evolution during heat treatment. The Mg phase and Si phase disappeared rapidly in the early stages of heat treatment. The amount of Al phase decreased rapidly in the beginning of heat treatment, but a small amount of Al still remained after further heat treatment. The Mg_2_Si phase formed gradually until ~4 min of heat treatment and remained at a relatively constant level during further heat treatment. The Al_3_Mg_2_ phase formed gradually until ~6 min of heat treatment and decreased slightly during further heat treatment. Interestingly, the Al_12_Mg_17_ phase formed rapidly within ~20 sec of heat treatment and almost disappeared at ~3 min. In conjunction with the kinetics of phase formation, the detailed phases of the end products were determined precisely by synchrotron X-ray diffraction. Type B contained Mg_2_Si, Al_3_Mg_2_, Al, Al_12_Mg_17_ (small amount), and Si phases. In contrast, Type C showed Mg_2_Si, Al_3_Mg_2_, Al, and Si phases, but the disappearance of Al_12_Mg_17_ was mostly confirmed.

The real-time characterization of phase formation revealed the following: Al_12_Mg_17_ and Al_3_Mg_2_ form first; Al atoms diffuse up toward the Mg layer and Mg atoms diffuse downwards; Mg_2_Si then forms; Mg atoms diffuse down toward the Al-Si layer and forms a solid solution with α-Al, and once over the solubility limit, Al_3_Mg_2_ begins to form; Al_12_Mg_17_ forms and disappears rapidly in the early stages of heat treatment; Al atoms diffuse toward the Mg layer to form Al_12_Mg_17_; and Al_12_Mg_17_ then decomposes and becomes a source of Mg to form Mg_2_Si. Therefore, the Mg-rich Al_12_Mg_17_ phase will be destroyed by Si; there is competition for Mg atoms in the formation of Mg-Si and Mg-Al bonds. The interaction of Mg-Si is much stronger than that of Al-Mg but the Si/Al ratio is approximately 1/9 which favors the formation of Al-Mg bonds at the early nano-metallurgy stages.

The above proposed chemical reactions of the Si-Al-Mg systems are consistent with thermochemistry calculations using the FactSage software^21^. Thermodynamically, Mg_2_Si, Al_3_Mg_2_, and Al_12_Mg_17_ will initially reach equilibrium just beneath the Mg layer. Indeed, as shown in Fig. 3, very few Si atoms manage to reach the Mg top layer in the as-grown sample. The presence of Mg_2_Si and Al_3_Mg_2_ was minimum, as shown in Fig. 4.

### Corrosion behavior

**Figure 5:**
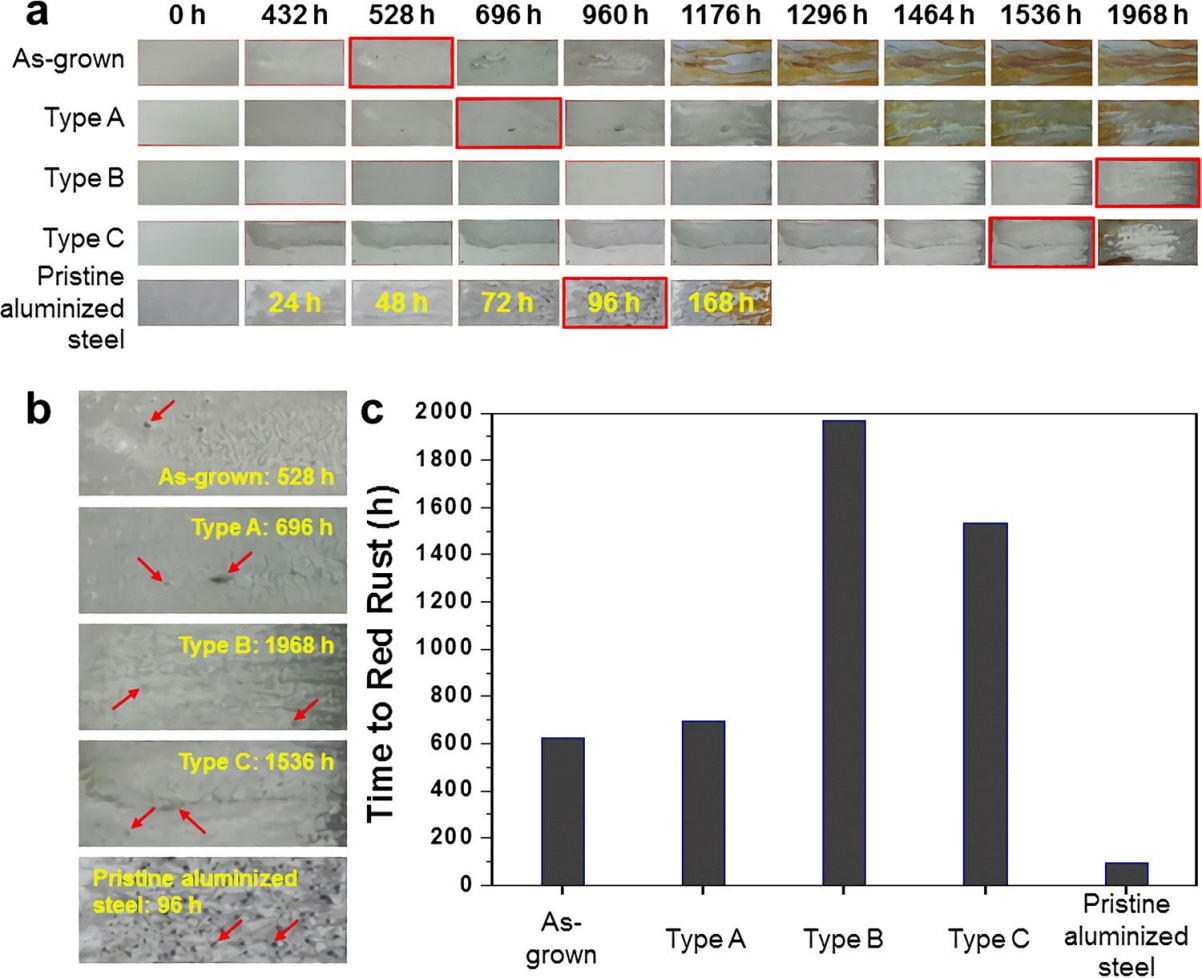
Results of the salt spray test for the samples with a 0.5 μm-thick Mg layer. The results of pristine aluminized steel without the Mg layer are included for comparison. (**a**) Optical photographs showing the appearance of red rust. (**b**) Enlarged optical photographs for the samples showing the first appearance of red rust. The red arrows indicate the red rust. (**c**) A bar graph was drawn based on the time when red rust began to appear.

Figure 5 presents photographs of the salt spray test for the samples with a 0.5 μm-thick Mg layer. The results of pristine aluminized steel without the Mg layer are included for comparison. Although the condition of the salt spray test was different from a real environment because the coupons under the test are exposed continuously to salt-containing mists without cyclic drying, a relative comparison of the corrosion resistance is effective. The pristine aluminized steel showed red rust on its surface after the 96 h test. In contrast, the appearance of red rust was delayed considerably: Type A 696 h, Type B 1,968 h and Type C 1,536 h. That is, the addition of Mg to aluminized steel increased the corrosion resistance almost 20 fold. In addition, Type B shows the best corrosion resistance, suggesting that careful control of the nano-metallurgy process is important. The salt spray test was performed repeatedly for samples with Mg layers with various thicknesses, and the trend of corrosion resistance was consistent. As an example, Supplementary Fig. 7 presents the results from the samples with a 1.0 μm-thick Mg layer.

The corrosion resistance of the cross-sectional samples was evaluated using galvanic measurements. As shown in Supplementary Fig. 8, the pristine aluminized steel has a very high potential of 800–850 mV. This was due to the stable, noble oxide layer, suggesting that the sacrificial effect is limited. In contrast, the Mg-coated aluminized steel has very low potential in the initial stages of galvanic corrosion, increasing slowly with increasing measurement time. This means that Mg-coated aluminized steel has an excellent sacrificial role. In particular, Type B showed the best sacrificial behavior. Accordingly, in combination with the results of the salt spray test, Type B showed the best corrosion resistance in terms of the surface and cross section.

## Discussion

### Role of Mg: Self-charge compensation

The corrosion resistance of aluminized steels was improved by the addition of Mg. The mechanism for the excellent corrosion resistance can be explained as follows. The corrosion resistance of regular eutectic aluminized steel is based on the protective ability of the oxides formed on the surface. The pristine aluminized layer was composed of 91 wt. % Al and 9 wt. % Si, eventually constructing a eutectic lamellar structure, as shown in Fig. 2. The lamellar structure exposed to air is likely to react promptly with oxygen, resulting in the formation of oxide phases, Al_2_O_3_ and SiO_2_. As a result, the surface of pristine aluminized steel is composed mainly of Al_2_O_3_ and partly of SiO_2_. Al_2_O_3_ is an excellent protection layer that can prevent oxygen or other corrosive elements from diffusing into the aluminized layer. This is the main source of the excellent corrosion resistance of aluminized steel. SiO_2_ is also an oxide phase with excellent corrosion resistance. What is important here is the presence of interfaces between the Al_2_O_3_ and SiO_2_ clusters due to the lamellar structure. These interfaces are exposed to air.

The Al_2_O_3_-SiO_2_ binary phase diagram shows an immiscibility gap, suggesting that Al_2_O_3_ and SiO_2_ clusters are immiscible with each other, i.e., they prefer to segregate themselves. Therefore, the weak Al-O-Si bonds in the interface between Al_2_O_3_ and SiO_2_ grains are potentially vulnerable to the penetration of oxygen and other corrosive elements.

**Figure 6:**
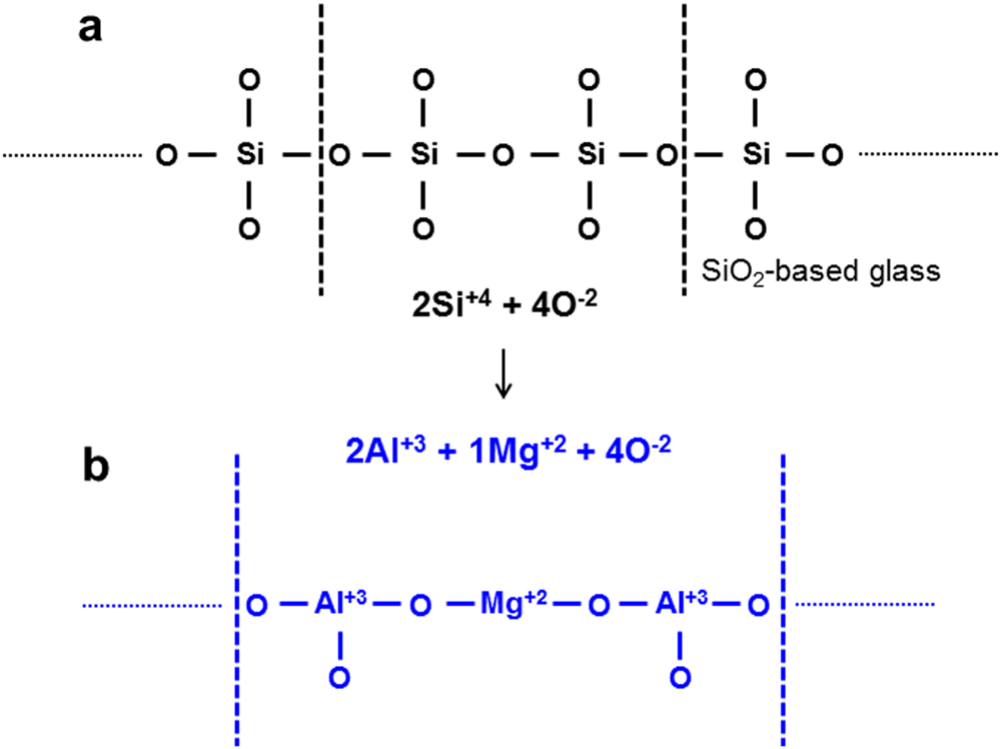
Schematic diagrams. (**a**) SiO_2_ is a glass former with a tetrahedral bond to oxygen. (**b**) Charge compensation of Si and Al ions by Mg addition, showing two Al^+3^ ions and one Mg^+2^ ion forming bonds to four O^−2^ ions while satisfying charge compensation, being equivalent to an MgAl_2_O_4_–SiO_2_ solid solution.

In this respect, making the immiscible interface more intimate is the key to enhancing the corrosion resistance of pristine aluminized steel. As shown schematically in Fig. 6a, SiO_2_ is a glass former with a tetrahedral bond to oxygen. In contrast, Al_2_O_3_ is a glass breaker (when forming a solution with SiO_2_) with a triangular bond to oxygen. This means that the addition of Al_2_O_3_ will weaken the glass network due to charge mismatch between the Si and Al ions, which is the origin of the immiscible interface between Al_2_O_3_ and SiO_2_. When an element that can compensate for the charge difference between Si and Al ions is supplied to the immiscible interfaces, they can be transformed to miscible ones. In other words, a third element is needed to overcome the charge difference between Al and Si in the oxide lattice to prevent the penetration of oxygen species from the environment to the alloy body.

**Figure 7:**
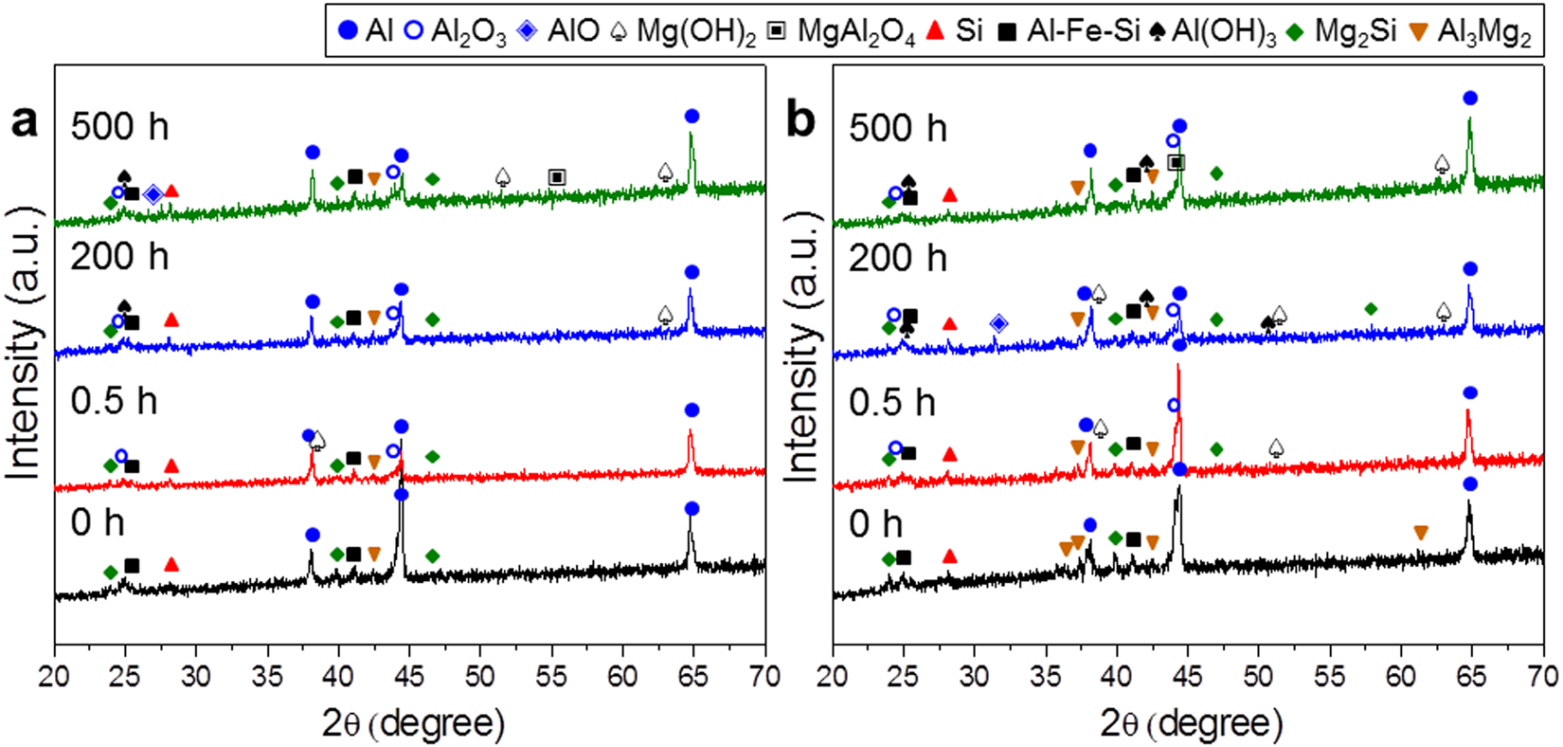
X-ray diffraction patterns taken from the Type B sample during the salt spray test. (**a**) Samples with a 0.5 μm-thick Mg layer. (**b**) Samples with a 1.0 μm-thick Mg layer.

As shown schematically in Fig. 6b, the addition of Mg to the Al-Si eutectic lamellar structure can compensate for the charge difference between Si and Al ions. Two Al^+3^ ions and one Mg^+2^ ion form bonds to four O^−2^ ions while satisfying the charge compensation. In terms of stoichiometry, this is equivalent to an MgAl_2_O_4_–SiO_2_ solid solution. The thermodynamic calculations using FactSage software^14^ revealed the formation of MgAl_2_O_4_ spinel, which is also supported by the XRD pattern of the corrosion products shown in Fig. 7. The corrosion products of the Type B sample were examined to determine if the spinel phase forms. As shown in Fig. 7, the XRD pattern and analysis of the chemical compositions revealed the formation of a spinel phase during the corrosion process. The miscible interfaces between Al_2_O_3_ and SiO_2_ will provide a more protective layer against corrosion.

**Figure 8:**
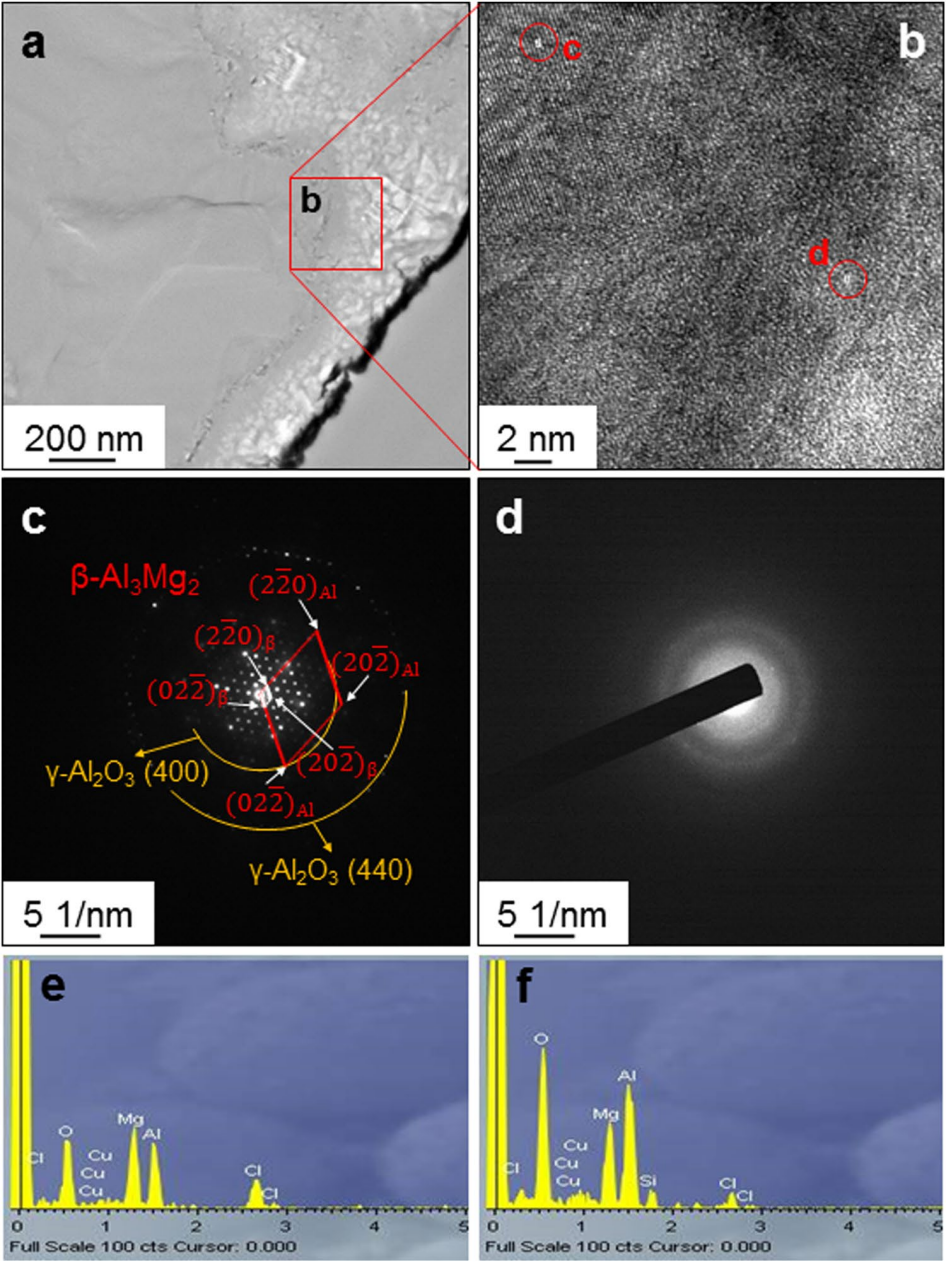
Results of high-resolution TEM analysis taken from the Type B sample with a 0.5 μm-thick Mg layer after the 100-hr salt spray test. (**a**) Bright-field low-magnification TEM image taken from the grain boundary region. (**b**) Enlarged image for the region of the open red square in a. Selected area diffraction patterns (**c,d**) and EDS Spectra (**e,f**) taken from the regions of the open red circle noted as “c” and “d”, respectively, in Fig. 8b.

**Figure 9:**
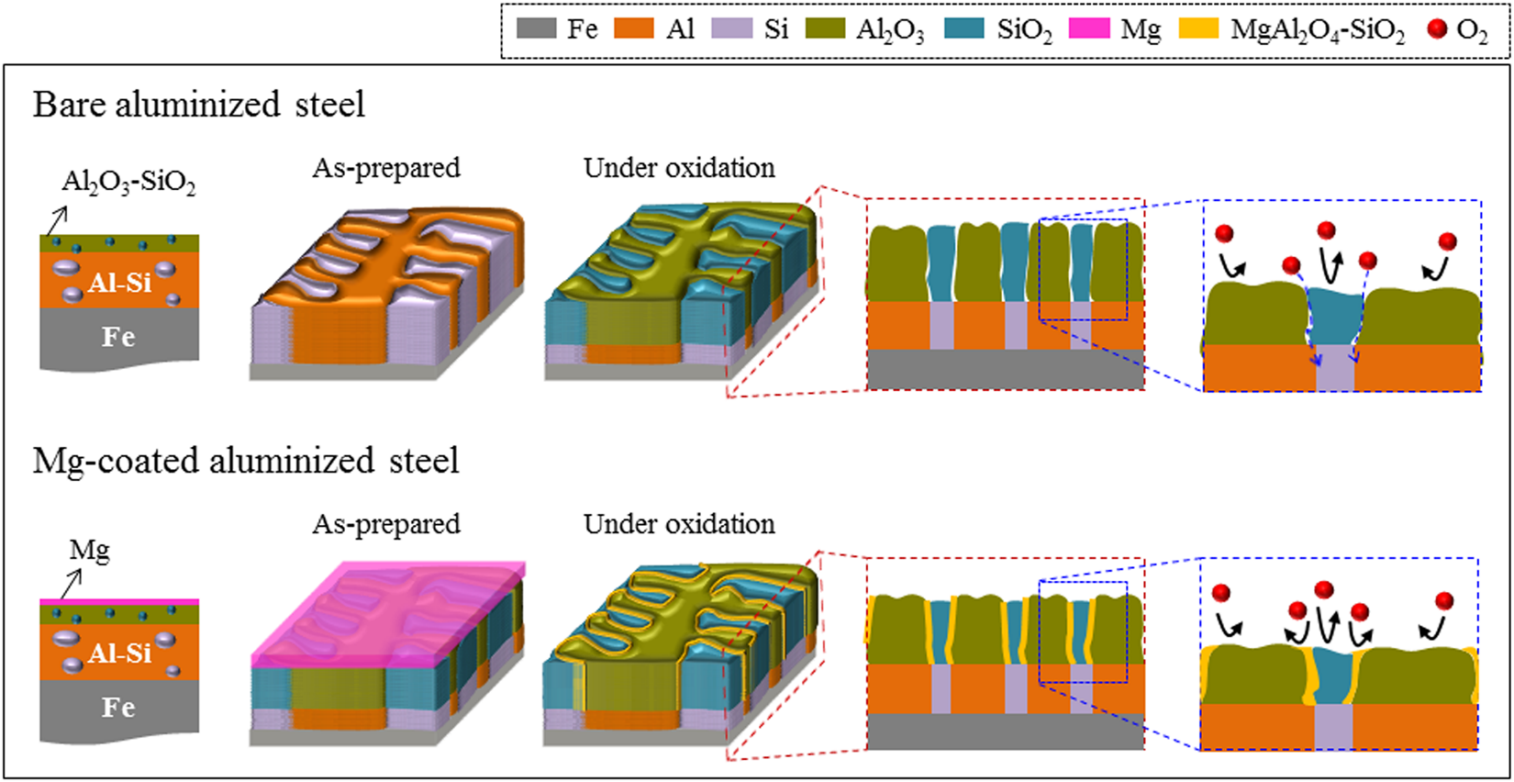
Schematic diagram showing the mechanism accounting for the super anticorrosion of Mg–coated aluminized steel. The pristine aluminized steel has immiscible interfaces between the Al_2_O_3_ and SiO_2_ clusters, through which oxygen species can penetrate easily (the upper figure). In contrast, in the case of Mg–coated aluminized steel, self-charge-compensated MgAl_2_O_4_-SiO_2_ phase will help seal the oxygen path between the clusters like a glue (the lower figure).

To further identify the formation of a spinel like charge self-compensated Si-Al-Mg-O amorphous phase, high-resolution TEM analysis was carried out for the Type B sample with a 0.5 μm-thick Mg layer after a 100-hr salt spray test; the results are presented in Fig. 8. Figure 8a presents the bright-field low-magnification TEM image taken from the area near a grain boundary. The open red square in Fig. 8a is enlarged as Fig. 8b. The open red circles noted as “c” and “d” in Fig. 8b correspond to areas of the grain interior and grain boundary, respectively. The selected area diffraction pattern, as shown in Fig. 8c, obtained from the area of the open red circle noted as “c” in Fig. 8b clearly show spotty patterns originating from Al_2_O_3_ and Al_3_Mg_2_. This demonstrates the crystalline phase of the eutectic Al part. In sharp contrast, the selected area diffraction pattern, as shown in Fig. 8d, obtained from the area of the open red circle noted as “d” in Fig. 8b displays a ring pattern, indicating a non-crystalline amorphous phase. Figure 8e,f show the EDS data taken from the areas of the open red circles noted as “c” and “d” in Fig. 8b, respectively. As evident, the crystalline area consists of Al, Mg, and O, whereas the amorphous area contains Al, Mg, O, and Si. Based on the selected area diffraction and EDS of the grain boundary area, it is reasonable to conclude that the Si-Al-Mg-O amorphous phase is present at the grain boundaries. EDS also revealed Cl from the NaCl solution used in the salt spray test and Cu from the TEM sample grids. Figure 9 presents a schematic diagram of the mechanism accounting for the super anticorrosion of Mg–coated aluminized steel.

In addition to the self-charge compensation by Mg addition, the formation of Mg_2_Si will play a sacrificial role in the corrosion of steel. The galvanic measurements shown in Supplementary Fig. 8 support the sacrificial role of Mg_2_Si, which in accordance with Tsuru *et al*.^16^.

### Controlled releasing of Mg

The remaining thing to understand is why Type B showed the best corrosion resistance. The key to the excellent corrosion resistance is the formation of good charge-compensating crystalline MgAl_2_O_4_ (Si solid solution) or amorphous Al-Si-Mg-O nanoclusters. According to the ternary Al_2_O_3_-SiO_2_-MgO phase diagram^22^, a zone is observed in the cordierite phase, where the liquidus isothermal line is almost parallel departing from the SiO_2_ corner to the MgO/Al_2_O_3_ = 1/2 ratio line. This self-charge compensated phase facilitates the compaction of the immiscible interfaces that will prevent oxygen ions passing from the air/liquid to beneath the eutectic lamellar Al-Si alloys.

On the other hand, the supply of Mg needs to be regulated to extend the effect of the miscible interfaces as long as possible. Here, the formation and growth of Mg_2_Si is very important. Mg_2_Si is quite stable. Besides being oxidized, once it forms, it will never transform to other phases. According to the *in-situ* XRD measurements, Type B contains α-Al, Al_3_Mg_2_ (β), Al_12_Mg_17_ (γ), and Mg_2_Si. Type C contains more Mg_2_Si and a negligible amount Al_12_Mg_17_ (γγ). In particular, a competition reaction occurs between Mg_2_Si and Al_12_Mg_17_ (γ) during heat treatment. Mg atoms in Al_12_Mg_17_ (γ) are likely to react with Si atoms, forming Mg_2_Si, which leads to the disappearance of Al_12_Mg_17_ (γ) and the formation of Mg_2_Si.

In Type B, there are three sources of Mg: α-Al, Al_3_Mg_2_ (β), and Al_12_Mg_17_ (γ). In contrast, only two sources of Mg are present in Type C: α-Al and Al_3_Mg_2_ (β). Mg_2_Si cannot be a source of Mg atoms because it is so stable and will not decompose. Therefore, the Mg supply is regulated longer in Type B than in Type C, meaning that longer corrosion resistance is realized in Type B. Careful control of phase evolution is essential to securing the maximized corrosion resistance.

Koch *et al*.^23^ observed the self-healing processes of a wax coating on plant surfaces, by direct measurements using atomic force microscopy (AFM). Voids in the wax coating were refilled by the formation of a new self-assembly wax film, from new wax molecules stored in the cells beneath the film. They also reported that this self-healing process is limited in some plants due to non-existing or low wax synthesis inside the cells. Therefore, for effective self-healing, it is important to (1) store (or quickly form) wax molecules in the cells beneath the damaged voids on the plant surface, and (2) to quickly supply the stored wax molecules in dynamic response to the formation of surface damage. Inspired by this bio-self-healing mechanism, it is important to look for opportunities to store and control the supply of Mg (the count-part of wax molecules in a plant) inside the coatings and release Mg in dynamic response to surface damage. Two “storages” were found for the original Mg atoms: Al_12_Mg_17_ (rich in Mg) and Al_3_Mg_2_ (rich in Al). For effective self-healing in the current metallic coatings, the formation of a spinel like charge self-compensated Si-Al-Mg-O phase is critical, which is the counterpart of the refilled wax film in a damaged plant surface. The following is proposed to mimic the self-healing of lotus leaves: (1) to make use of the wax refilling mechanism to form spinel-like Si-Al-Mg-O coating for surface self-healing; (2) to form MgAl_2_O_4_ spinel by controlled Mg diffusion, similar to the new wax molecules in the cells, via the use of a two-stage Mg storage (Al_12_Mg_17_ and Al_3_Mg_2_) that facilitates the control of Mg migration; and (3) to facilitate the control of Mg_2_Si formation using the above two-stage Mg storage, finally forming the desired Si-Al-Mg-O glue from MgAl_2_O_4_-Mg_2_Si systems. Therefore, like the bio-inspired process in lotus leaves, which self-supplies a high surface energy material to a damaged surface, a solution for enhancing the corrosion resistance may be to optimize the supply of Mg.

## Methods

### Hot-dip aluminized steel

Commercially available aluminized steel (POSCO C&C) was used as the substrate. Cold-rolled steel sheets, 0.4 mm in thickness, with the composition of Fe–0.08 wt. % C–0.45 wt. % Mn–0.03 wt. % P–0.03 wt. % S, underwent a hot-dip aluminizing process. The thickness and composition of the resulting aluminized layer was approximately 5 μm and Al–9 wt. % Si, respectively.

### Deposition of the Mg layer and subsequent heat treatment

Mg layers of various thicknesses (0.3, 0.5, and 1.0 μm) were deposited on the aluminized steel substrates by magnetron sputtering using a high purity (99.99%) Mg target with a thickness and size of 10 mm and 800 × 120 mm^2^ (rectangular shape), respectively. Before being loaded in the growth chamber, the hot-dip aluminized samples were cleaned by degreasing in an alkaline solution, and sequential ultrasonication in acetone and alcohol for 5 min each. To remove the natural hydroxide and oxide, the substrate surface was exposed to an Ar ion beam of 3 kV and 450 mA in the growth chamber for 5 min.

The sputtering conditions adopted were as follows: chamber pressure, input power, gas flow rate, gas ambient, and target to substrate distance of 10 mTorr, 2.5 kW, 80 sccm, argon, and 70 mm, respectively. No intentional substrate heating was applied. The resulting deposition rate was estimated to be approximately 50 nm/min. After the sputter deposition of Mg, the samples underwent heat treatment under the following conditions: heating temperature of 400 °C; duration of 2, 5 and 10 min; ambient atmosphere of 1 atm N_2_; and cooling in air. In this study, the samples were classified with regard to the heat treatment time; the 2, 5 and 10 min heat-treated samples were designated as Types A, B, and C, respectively.

### Material characterization

The microstructure of the samples was examined by field-emission scanning electron microscopy (FE-SEM, Hitachi, S-4300SE) and transmission electron microscopy (TEM, JEOL, ARM200F). The bulk and surface chemistries were characterized simultaneously by energy-dispersive X-ray spectroscopy (EDS, Oxford INCA energy) and electron probe micro analysis (EPMA, Shimadzu, EPMA-1600). Phase identification was performed by X-ray diffraction (XRD, D-MAX, 2500-VPC) using CuKα radiation (λ = 1.541 Å). The water contact angles were measured using a custom-made contact angle analyzer at room temperature with a 5 μl droplet of deionized water.

To examine the real-time phase transformation of the Mg-coated aluminized samples during heat treatment, synchrotron X-ray scattering measurements were carried out at Beamline D at the Pohang Light Source (PLS) in Korea. The incident X-rays were focused vertically using a mirror, and monochromatized to a wavelength of 1.757 Å using a double-crystal Si (1 1 1) monochromator. The second sagittal crystal of the monochromator also focused the X-rays horizontally. The momentum transfer resolution was controlled by two pairs of slits on the detector arm, which were set to 0.001 Å^−1^. The as-prepared aluminized steel sheet was placed into the chamber that was basically identical to the heat treatment furnace. During heat treatment, the specimens were characterized by a fixed-theta mode using a 1-dimensional strip X-ray detector.

### Corrosion test

Pristine, Mg-coated, and heat-treated aluminized steel samples, 5 × 10 cm^2^ in size, underwent a salt spray test, which is one of the best known accelerated corrosion tests, in accordance with ASTM B117. With this test, corrosion-resistance information for the samples could be provided on a relative basis. Spraying a 5 wt. % NaCl aqueous solution resulted in uniform droplets on the specimens suspended ~30° from the vertical in the test chamber. The exposure zone of the salt spray chamber was maintained at 35 °C. The salt-rich condensates are likely to attack the surface of the samples, and were examined at 24 h intervals.

A galvanic corrosion test was carried out to evaluate the sacrificial anode effect of the prepared coating samples against a steel sheet (Fe) in a corrosive environment. Under the galvanic corrosion test, the exposed surface areas of the coating sample and steel sheet (Fe) as the galvanic couple were 1 cm^2^ and 25 cm^2^, respectively. The galvanic corrosion current and potential were measured in a 3% NaCl aqueous solution at room temperature using a potentiostat (USA, Garmy Interface 1000). At this time, the mixed potential of the galvanic couple was measured using a silver-silver chloride reference electrode (SSCE, Ag/AgCl electrode). The distance of the galvanic couple between the coating sample and steel sheet was kept at 1 cm.

### Data availability

All data supporting the findings of this study are available within the article and its Supplementary Information file, or are available from the corresponding authors upon request.

## Electronic supplementary material

Supplementary Information
