# Supplementary material for: Super anticorrosion of aluminized steel by a controlled Mg supply
Source: Sci Rep. 2018 Feb 28;8:3760. doi: 10.1038/s41598-018-22097-z (PMC5830645; doi:10.1038/s41598-018-22097-z)
Supplement: Supplementary file 1 — Supplementary Information [file 41598_2018_22097_MOESM1_ESM.pdf]

Supplementary Information

Super anticorrosion of aluminized steel by a controlled Mg supply

Jae In Jeong[1,*], Ji Hoon Yang[1], Jae Hun Jung[1], Kyung Hwang Lee[1], Hye Jeong Kim[1], Yong Hwa Jung[2], Tae Yeob Kim[2], Myeong Hoon Lee[3], Sung Hwa Hwang[3], Ping Wu[4,*], Jae-Hun Kim[5] & Sang Sub Kim[5,*]

[1]*Materials Solution Research Group, Research Institute of Industrial Science & Technology, 67 Cheongam-ro, Nam-gu, Pohang 37673, Republic of Korea*
[2]*Posco Smart Coating Technology-Dry Coating Project Dept., POSCO Gwangyang Research Lab., 20-26 Pokposarang-gil, Gwangyang 57807, Republic of Korea*
[3]*Division of Marine Engineering, Korea Maritime & Ocean University, 727 Taejong-ro, Yeongdo-gu, Busan 49112, Republic of Korea*
[4]*Entropic Interface Group, Singapore University of Technology & Design, Singapore 487372, Singapore.*
[5]*Department of Materials Science and Engineering, Inha University, Incheon 22212, Republic of Korea.*

Correspondence and requests for materials should be addressed to Jae In Jeong (jijeong@rist.re.kr), Ping Wu (wuping@sutd.edu.sg) or Sang Sub Kim (sangsub@inha.ac.kr).

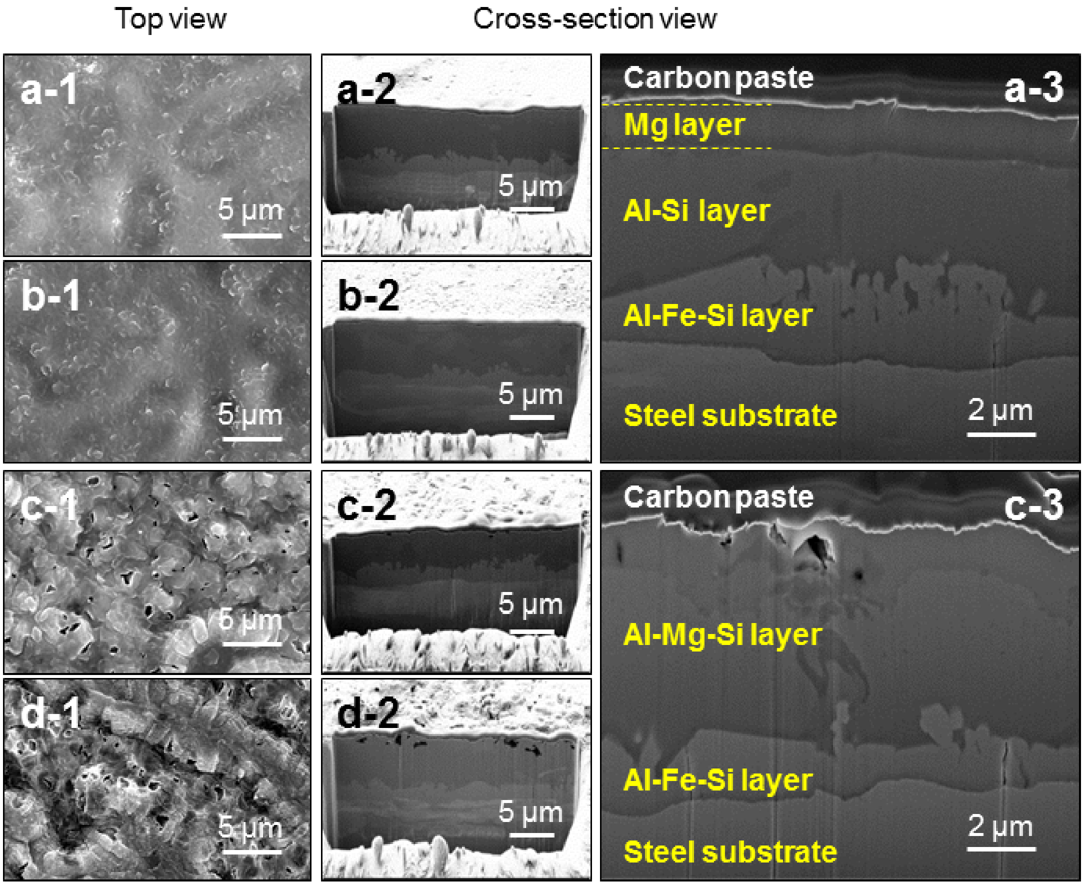

Supplementary Fig. 1 Scanning electron micrographs of the as-grown and heat-treated samples with a 1.0 µm-thick Mg layer: Top view and cross-section view images of **a-1** and **a-2** as-grown, **b-1** and **b-2** Type A, **c-1** and **c-2** Type B, and **d-1** and **d-2** Type C samples, respectively. **a-3** and **c-3** are magnified images for **a-2** and **c-2**, respectively. The carbon paste was used as a conducting layer for observation.

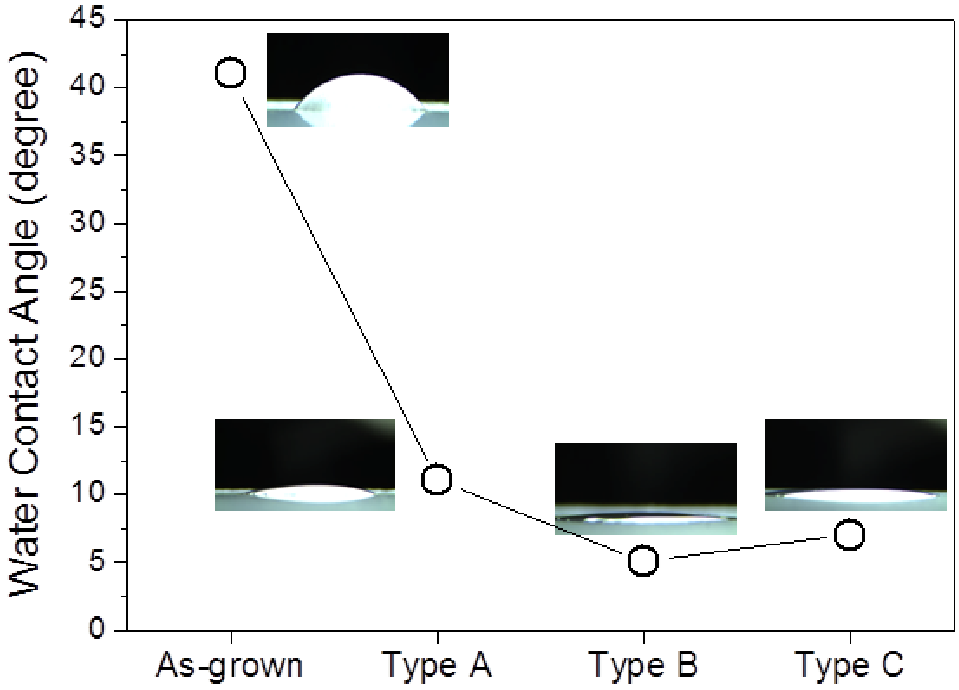

Supplementary Fig. 2 Contact angles of water droplets on the 4 surfaces with a 1.0 μm-thick Mg layer; as-grown, Type A, Type B, and Type C.

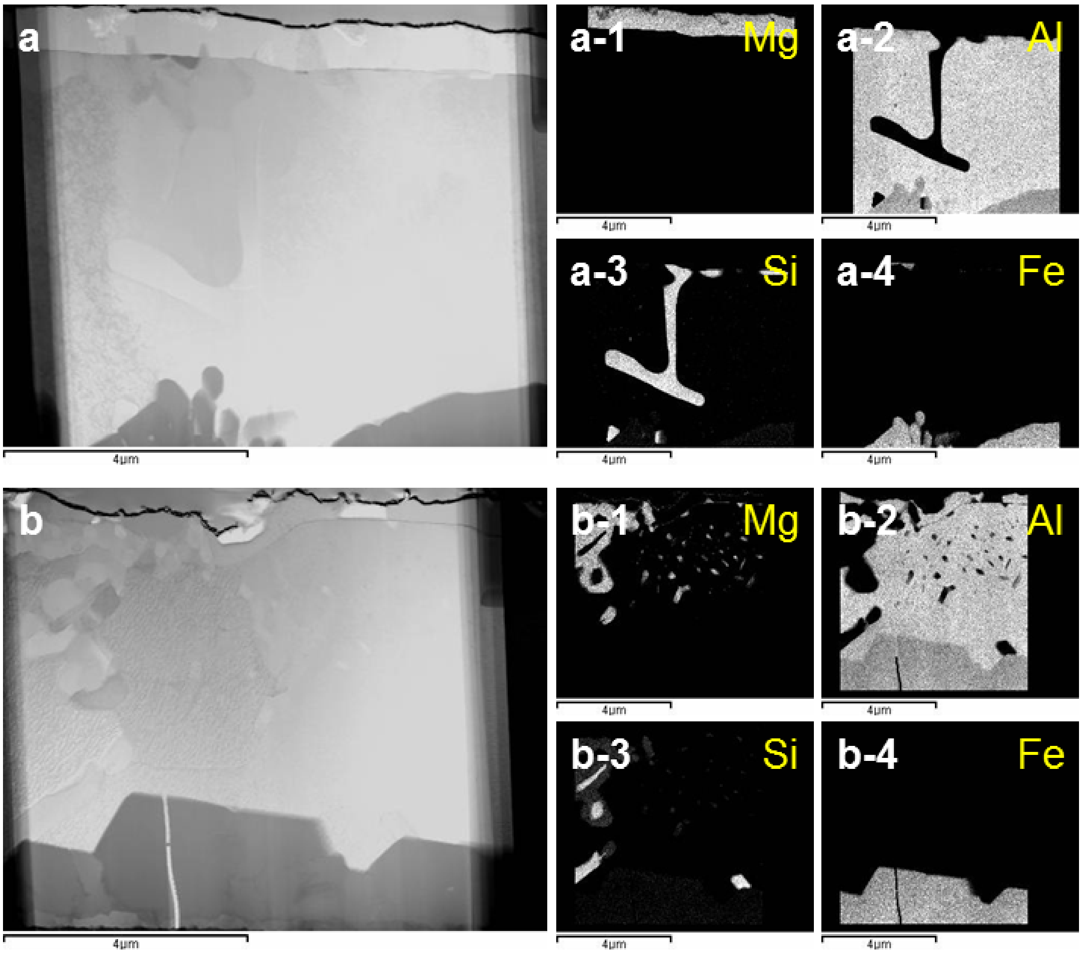

Supplementary Fig. 3 Bright-field transmission electron micrographs of **a** as-grown and **b** heat-treated Type C samples with a 0.5 μm-thick Mg layer. The corresponding elemental maps for Mg, Al, Si, and Fe are also presented.

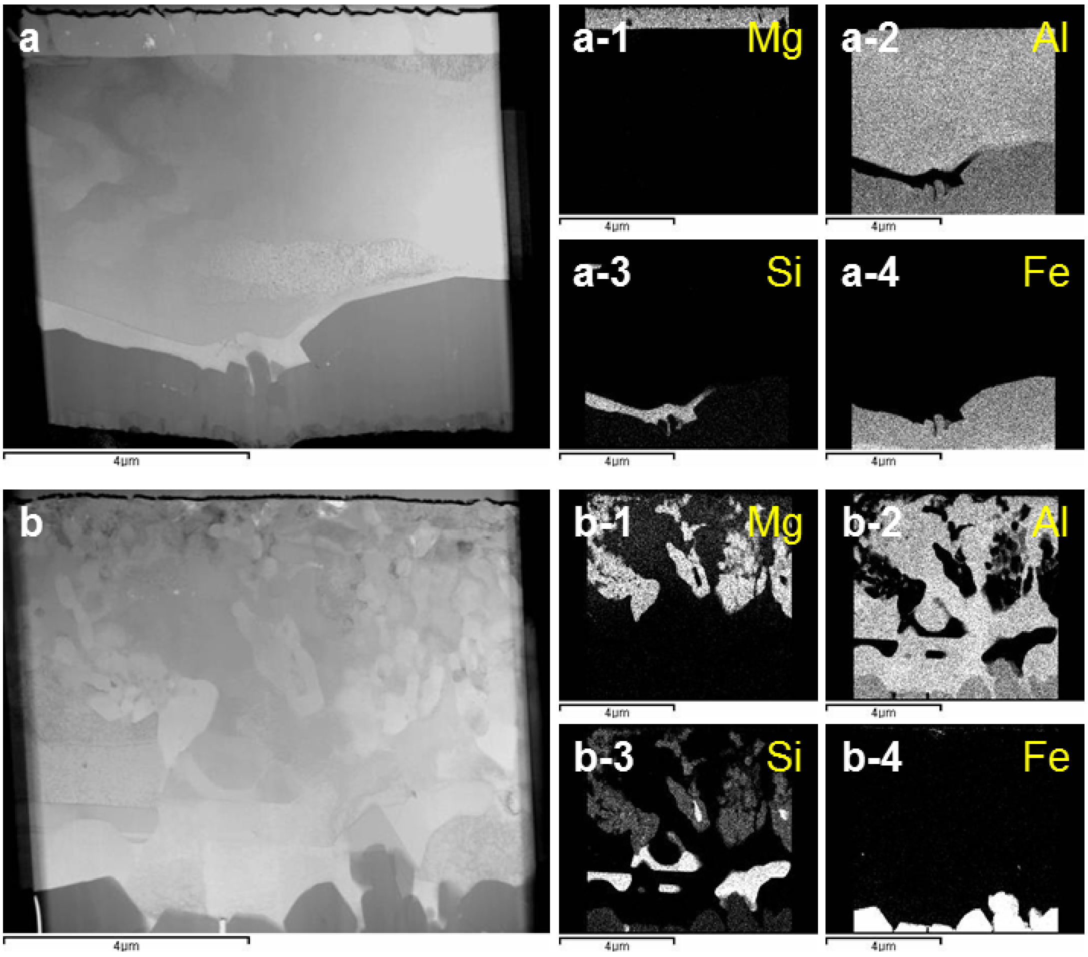

Supplementary Fig. 4 Bright-field transmission electron micrographs of **a** as-grown and **b** heat-treated Type C samples with a 1.0 μm-thick Mg layer. The corresponding elemental maps for Mg, Al, Si, and Fe are also presented.

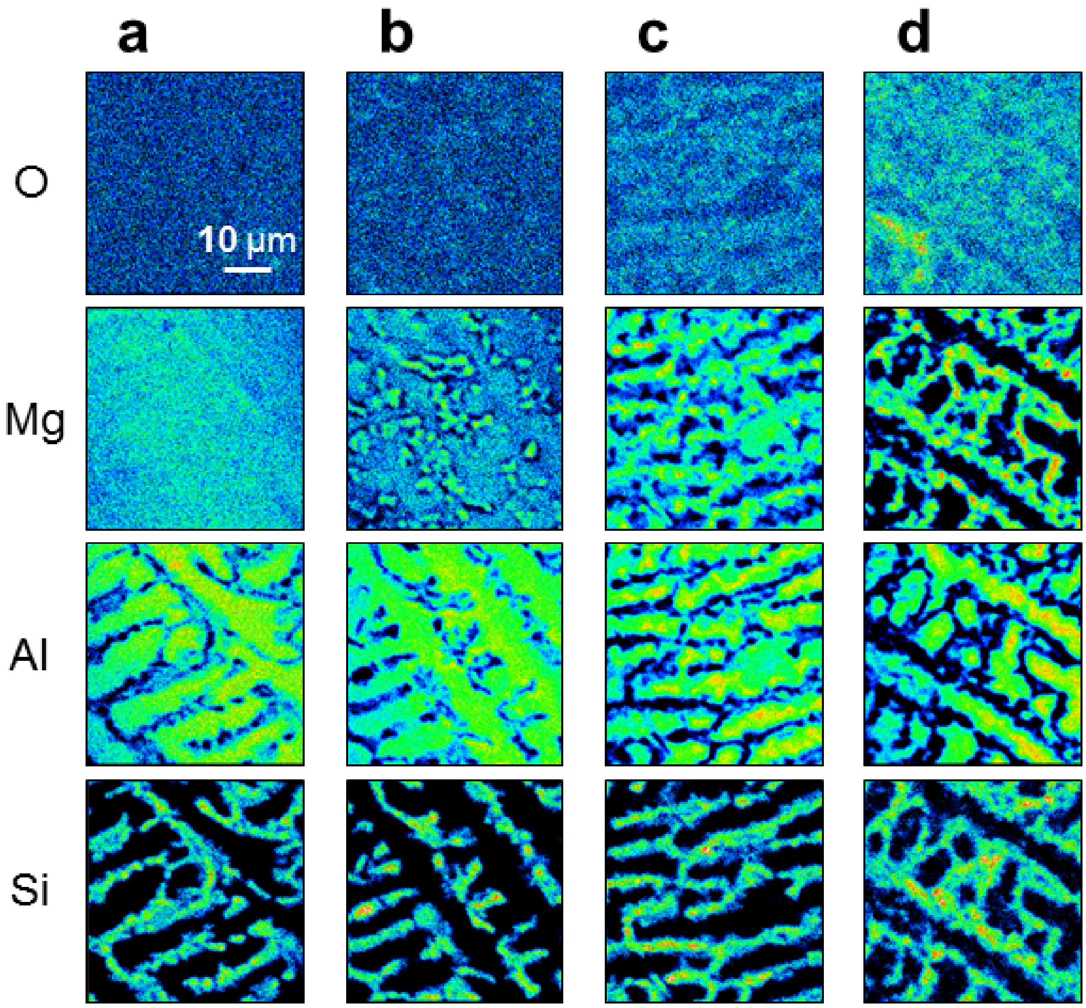

Supplementary Fig. 5 Top-view elemental maps by electron probe microanalysis for **a** as-grown, **b** Type A, **c** Type B, and **d** Type C samples a 0.3 μm-thick Mg layer.

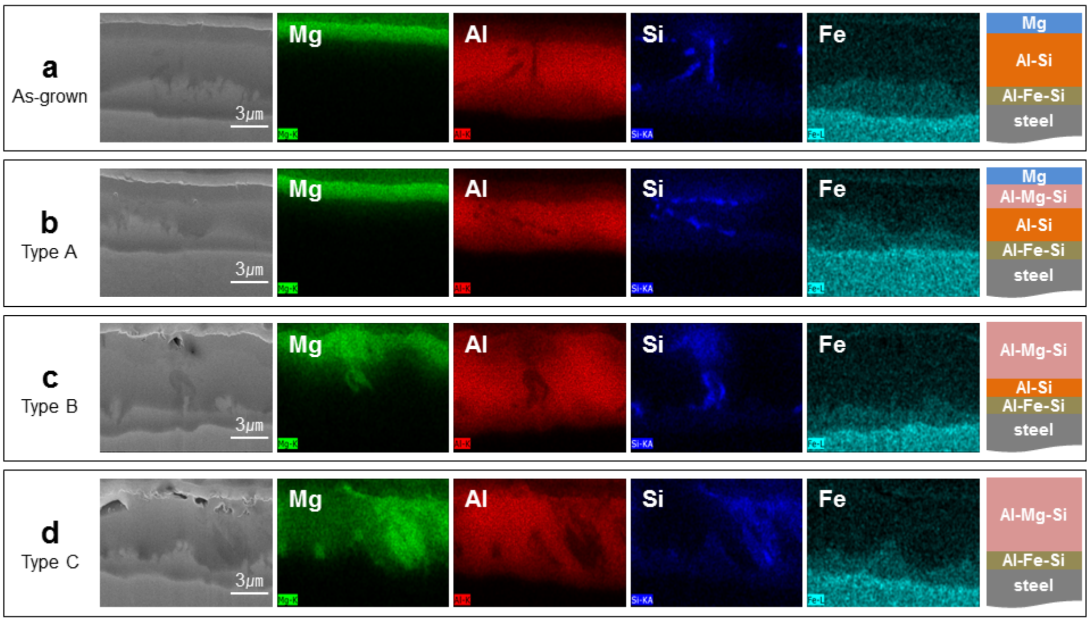

Supplementary Fig. 6 Cross-section view elemental maps by electron probe microanalysis for **a** as-grown, **b** Type A, **c** Type B, and **d** Type C samples with a 1.0 μm-thick Mg layer.

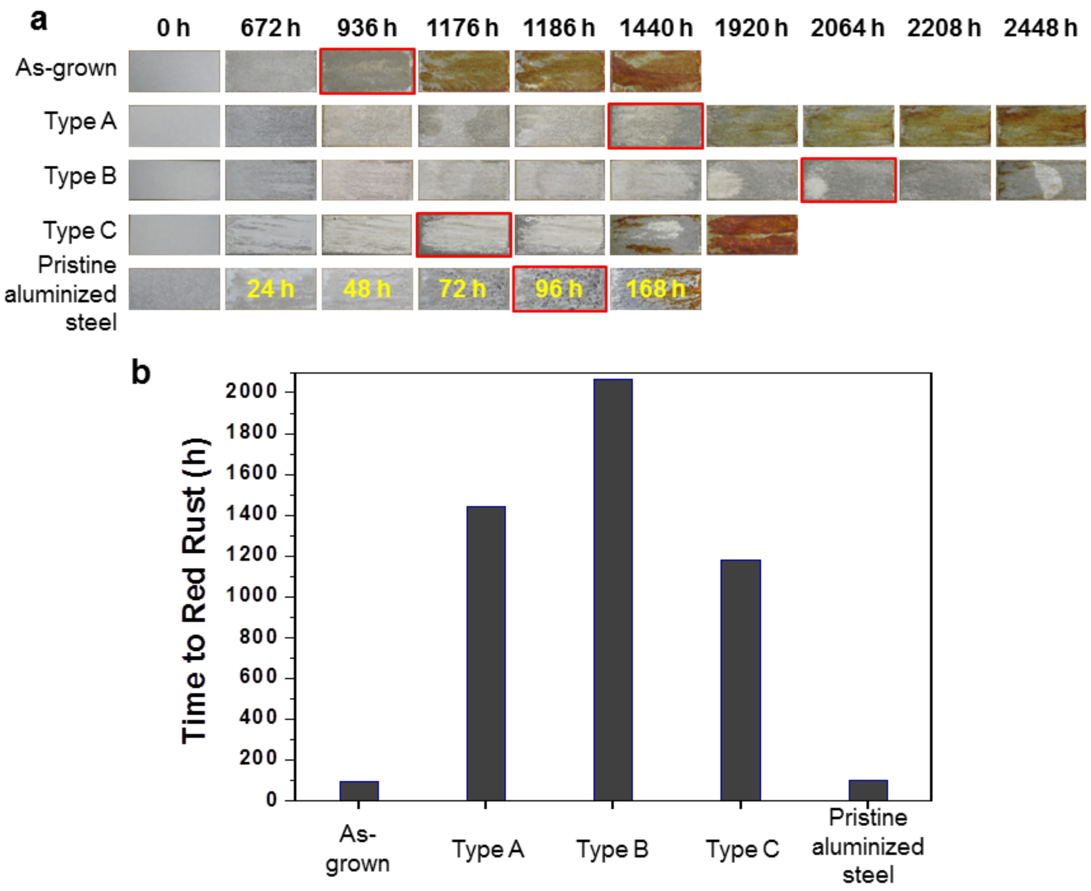

Supplementary Fig. 7 Results of the salt spray test for the samples with a 1.0 μm-thick Mg layer. For comparison, the results of regular aluminized steel without the Mg layer are included. **a** Optical photographs showing the appearance of red rusts. **b** A bar graph was drawn based on the time when red rust began to appear.

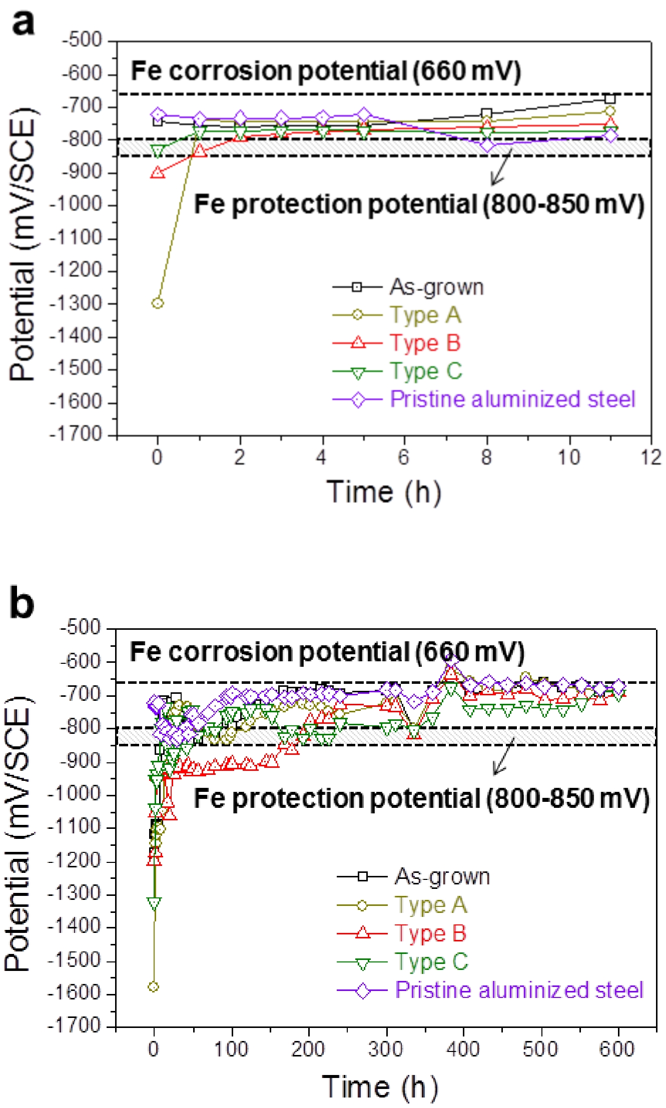

Supplementary Fig. 8 Results of galvanic measurements. The results taken from pristine aluminized steel are included for comparison. **a** Samples with a 0.5 µm-thick Mg layer. **b** Samples with a 1.0 µm-thick Mg layer.
